# Supplementary material for: Deletion of MGF505-2R Gene Activates the cGAS-STING Pathway Leading to Attenuation and Protection against Virulent African Swine Fever Virus
Source: Vaccines (Basel). 2024 Apr 11;12(4):407. doi: 10.3390/vaccines12040407 (PMC11054455; doi:10.3390/vaccines12040407)
Supplement: Supplementary file 1 [file vaccines-12-00407-s001.zip › vaccines-2946519-supplementary.pdf]

Table S1. Clinical signs score index, based on [44].

Temperature	0	<39
	1	39.0 < to < 39.5
	2	39.5 ≤ to < 40
	3	40.0 to ≤ 40.5
	4	40.6 to ≤ 41
	5	<39, >41
Inappetence	0	Normal
	1	Reduced eating
	4	Only picking at food
	6	Not eating
Recumbancy	0	Normal
	1	Lethargic
	2	Get up only when touched
	4	Slow to get up when touched
	6	Remain recumbent when touched
Skin hemorrhage	0	Normal
	1	Hemorrhagic areas on ears and body
	3	Generalized hemorrhage all over body
Joint swelling	0	Normal
	1	A joint swelling
	4	Severe swelling with difficulty walking
Labored breathing and/or coughing	0	Normal
	1	Mild
	3	Severe
Ocular discharge	0	Normal
	1	Gummed up eyes
Diarrhea	0	Normal
	1	Diarrhea
	4	Bloody diarrhea
Urine	4	Blood urine
Vomiting	4	Vomiting

Table S2. Clinical sign score of vaccine immunized pigs in Animal Experiment I.

dpv	Clinical sign score	
	Ave	SD
0dpv	2.8	1.0
1dpv	2.8	1.5
2dpv	2.5	1.9
3dpv	0.3	0.5
4dpv	2.3	1.3
5dpv	1.3	0.5
6dpv	2.8	1.5
7dpv	1.0	0.8
8dpv	1.8	0.5
9dpv	0.0	0.0
10dpv	2.0	0.8
11dpv	3.0	2.0
12dpv	2.5	1.7
13dpv	2.0	0.8
14dpv	1.8	0.5
15dpv	1.8	0.5
16dpv	1.3	1.0
17dpv	2.3	1.3
18dpv	0.0	0.0
19dpv	1.8	0.5
20dpv	2.0	0.8
21dpv	0.0	0.0
22dpv	0.0	0.0
23dpv	0.0	0.0
24dpv	0.0	0.0
25dpv	0.0	0.0
26dpv	0.0	0.0
27dpv	0.0	0.0
28dpv	2.0	0.8
